# Silent Echoes: A Case Report of Wernicke Encephalopathy's Unheard Voice

**DOI:** 10.7759/cureus.52151

**Published:** 2024-01-12

**Authors:** Stefan Gafoor, Raheem Robertson

**Affiliations:** 1 Graduate Medical Education, Piedmont Athens Regional, Athens, USA

**Keywords:** alcohol use, thiamine defecincy, wernicke-korsakoff syndrome, non-alcoholic wernicke's encephalopathy, wernicke’s

## Abstract

Wernicke encephalopathy (WE) is an acute neuropsychiatric emergency that is caused by a deficiency in vitamin B1 (thiamine). This condition is most commonly seen in patients with alcohol use disorder; however, patients with other disorders of severe malnourishment are also at increased risk. In severe cases, this disease may be followed by Korsakoff's psychosis and even death.

We present a case of a 64-year-old African American female with a history of alcohol use disorder who presented to the emergency department on account of an acute confusional state. Neurological examination revealed right beating nystagmus on the left gaze and a wide-based gait. Initial laboratory work-up was unrevealing; however, magnetic resonance imaging (MRI) of the brain demonstrated an abnormal T2 fluid-attenuated inversion recovery (FLAIR) signal involving the bilateral mammillary bodies and surrounding lateral ventricles that extended into the periaqueductal parenchyma. The patient was admitted to the neurology unit, and high-dose intravenous thiamine was commenced. During hospitalization, the patient's confusion improved and they were subsequently discharged with oral thiamine.

The spectrum of severity of WE is wide, ranging from fatal disease and can lead to permanent brain damage or even Korsakoff syndrome, characterized by severe memory loss and confabulation. The diagnosis is mainly clinical and based on the presence of symptoms in the classic triad of mental status change, oculomotor abnormality, and ataxia. This triad is only present in about 10% of cases, making the diagnosis very challenging. Laboratory testing can assist in making the diagnosis, but it is not always reliable or available. In situations of clinical uncertainty, imaging may also be used to support diagnosis or rule out other differentials. The mainstay of treatment is with high-dose parenteral thiamine.

## Introduction

Wernicke encephalopathy (WE) is a severe and potentially reversible neuropsychiatric disorder caused by a deficiency in thiamine. WE is characterized by the classic clinical triad of acute mental confusion, ataxia, and ophthalmoplegia [1,2]. This typical appearance is seen in only one-third of the cases. Autopsy studies show an estimated prevalence of 0.4% through 2.8 % with Australia having the highest prevalence in the Western world (1.1 % through 2.8%) [3,4]. Interestingly, the prevalence in patients with alcohol use disorder is staggeringly higher at 12.5%. WE may be followed in its acute phase by severe amnestic deficits, Korsakoff's psychosis, and even death if left untreated. Herein, we describe a case of an acute confusional state in a patient with a history of alcohol use disorder.

## Case presentation

A 64-year-old African American female with a 22-year history of alcohol use disorder presented to the emergency department (ED) on account of acute confusion of a day's duration. The patient was last seen well a day before her presentation. On the day of the presentation, the patient was reported to be confused and was described as being "spaced out". There were no preceding illnesses, trauma, or acute stressors, the patient was not on any prescription or over-the-counter medications and her last alcoholic beverage was one day before her presentation.

The patient's vital signs were within normal limits in the ED. On general inspection, she appeared disheveled, and in no apparent distress. On examination, the patient was confused and oriented only to self, mini-cog test score of 0 points. No tremors were noted; however, there was right beating nystagmus on the extreme left gaze, and gait was noted to be wide-based. The remaining neurological examinations including tone, power, and reflexes were within normal limits, as well as the remainder of her physical examination.

**Figure 1:**
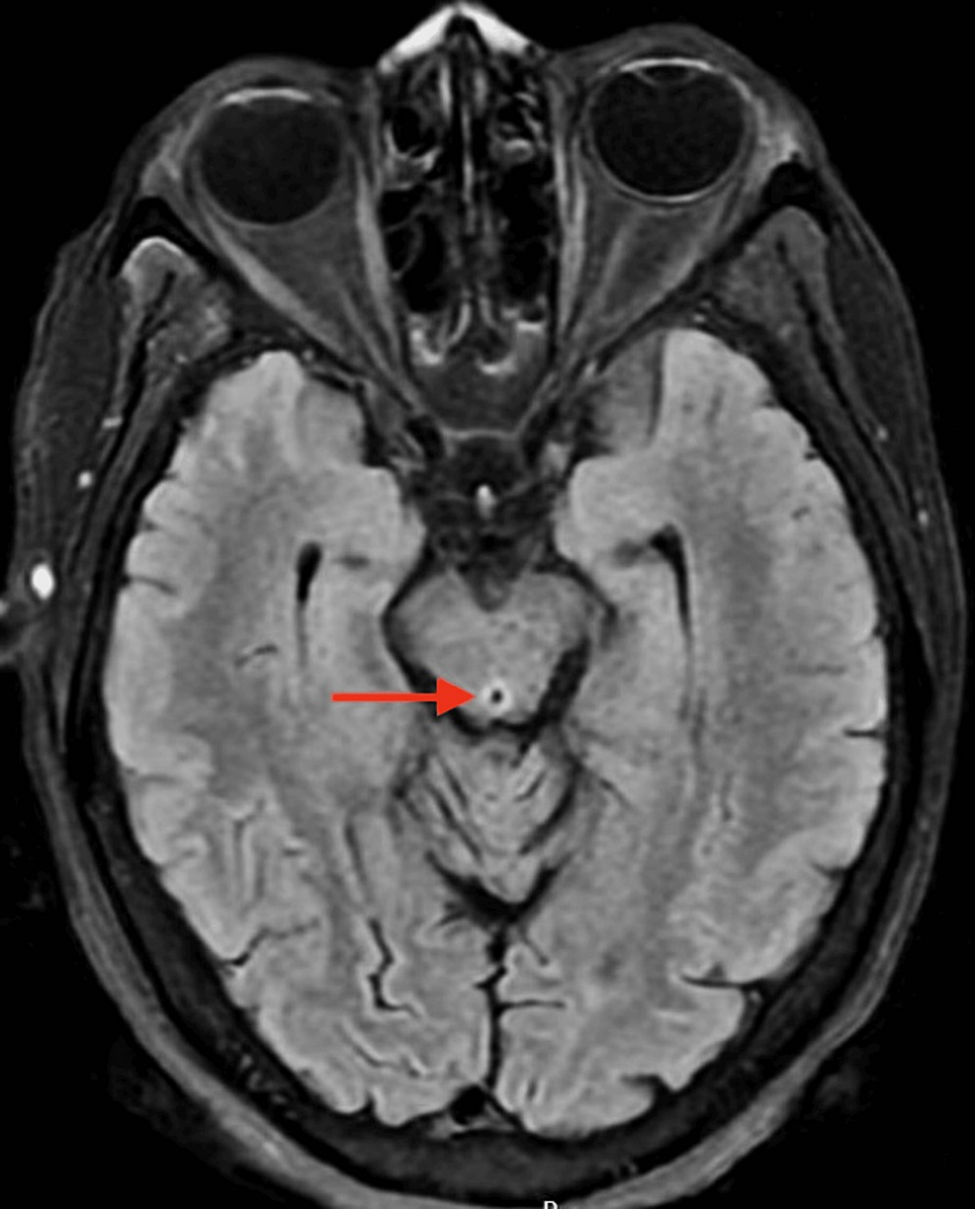
Non-contrast MRI, axial view demonstrating an increased T2 FLAIR signal in the periaqueductal grey matter. FLAIR: Fluid-attenuated inversion recovery

**Figure 2:**
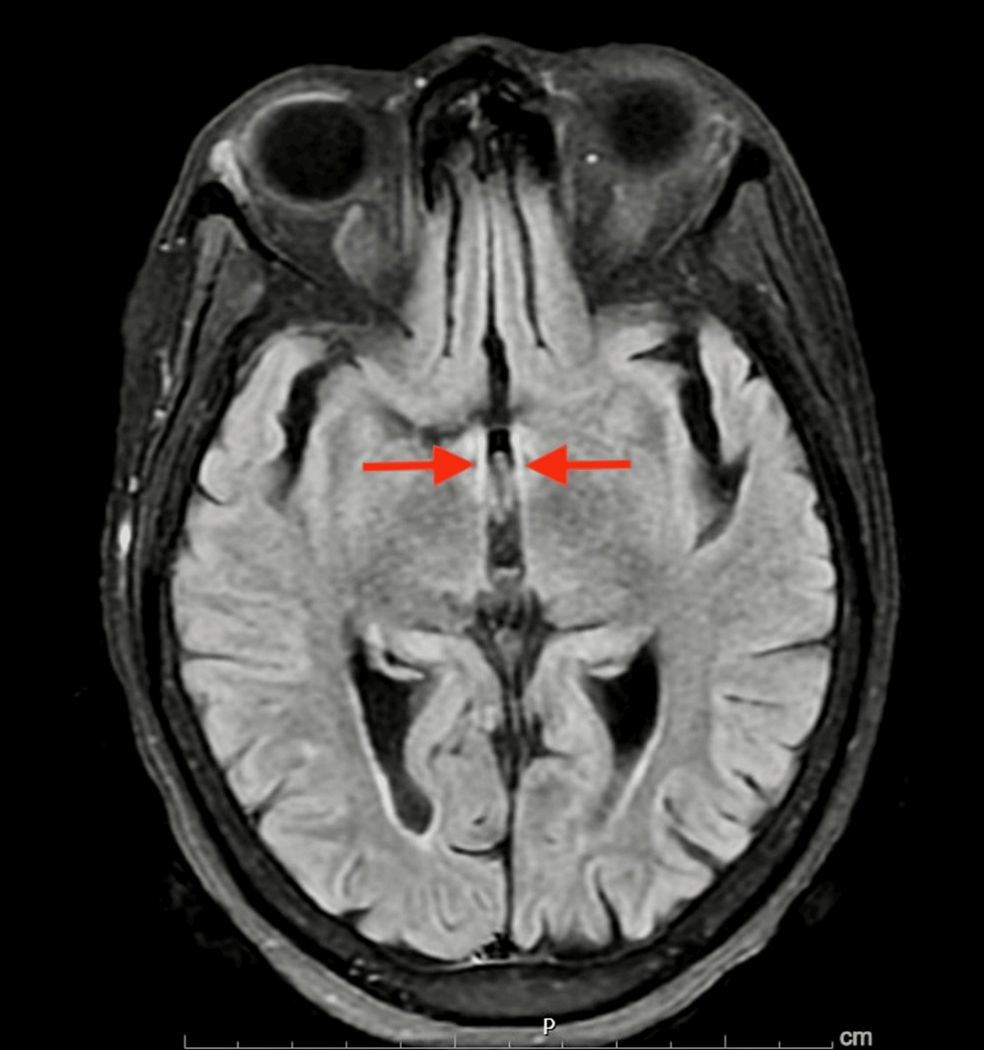
Non-contrast MRI, axial view showing increased T2 FLAIR signal affecting the bilateral mammillothalamic tracts (red arrows). FLAIR: Fluid-attenuated inversion recovery

**Figure 3:**
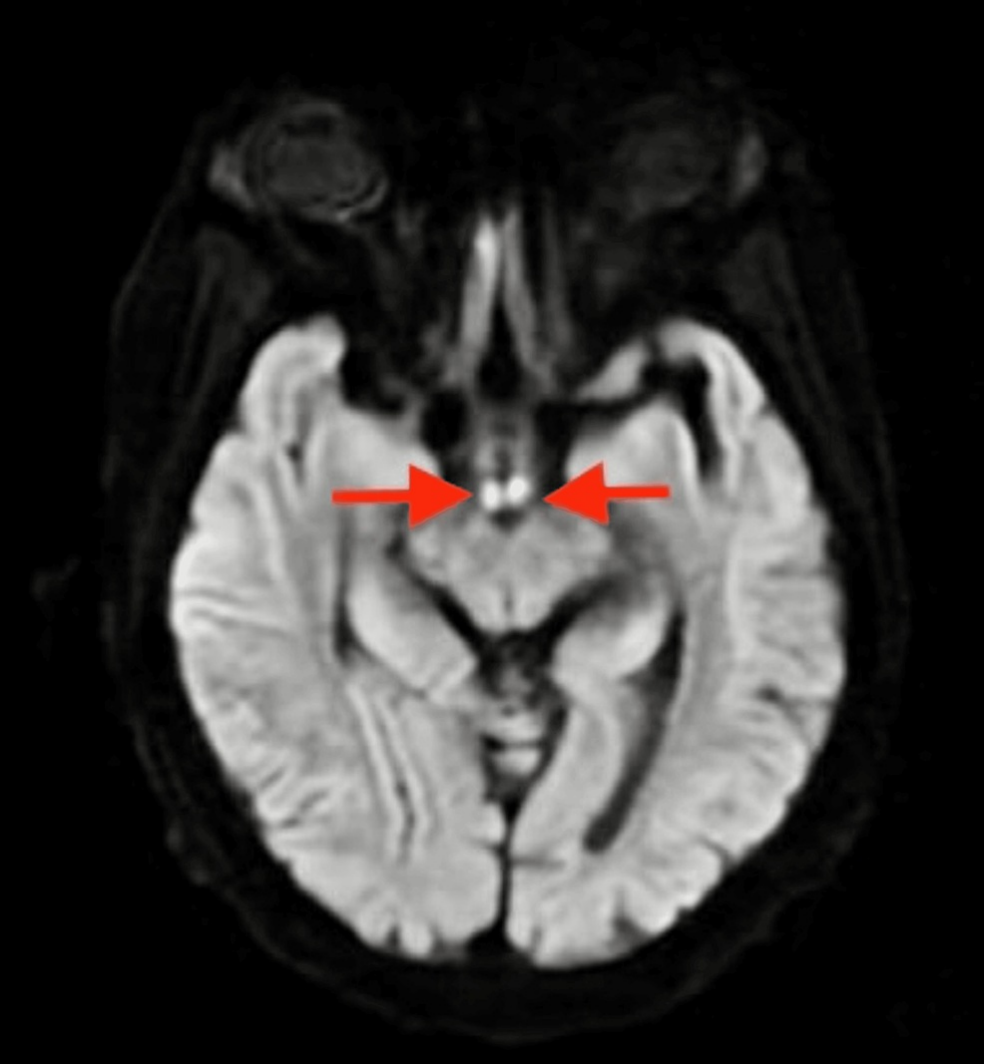
Non-contrasted MRI, axial view, DWI highlighting bright areas of high signal intensity affecting the mammillary bodies bilaterally (red arrows). DWI: Diffusion-weighted imaging

Initial laboratory work-up with complete blood count, and complete metabolic panel, revealed hypokalemia with a potassium level of 3.2 mEq/L, the remainder of values were normal, the urinary drug screen was negative, and the serum alcohol level was undetectable. Imaging done with magnetic resonance imaging of the brain (non-contrast) showed an abnormal transverse relaxation time (T2), fluid-attenuated inversion recovery (FLAIR) signal involving the periaqueductal grey matter (Figure 1), surrounding lateral ventricles, and mammillothalamic tracts (Figure 2), as well as a high signal intensity affecting the bilateral mammillary bodies as seen on diffusion-weighted imaging (DWI) (Figure 3). The patient was diagnosed with presumable WE and was admitted to the neurology unit for high-dose parenteral thiamine treatment (500 milligrams three times daily). The serum thiamine level obtained on admission resulted in three days later as < 6 nmol/L (normal 70-179 nmol/L). On day three of hospitalization, the patient's confusion had improved, and she ultimately completed seven days of high-dose parenteral thiamine which was then switched to an oral formulation. 

## Discussion

The typical description of WE as described by Carl Wernicke in the year 1881 is by a classic triad of mental status change, oculomotor abnormality, and gait ataxia [1,2]. The prevalence from some autopsy studies was 1.1%-2.8% [3,4]. The most common presenting symptom is a mental status change, which is present in up to 82% of cases; however, patients may also initially present with non-specific symptoms such as headaches, irritability, gastrointestinal upsets, and fatigue [5,6]. Only about 16-20% of cases present with all three features of WE described above as seen in the case presentation, our patient had all three manifestations of the condition [7]. The foremost etiology of WE is thiamine deficiency. 

Thiamine deficiency has many causes of which chronic alcohol use is a major contributor [5,8]. However, it is important to highlight non-alcohol-related causes such as malnutrition from other conditions (Table 1).

**Table 1:** Non-alcohol-related causes of Wernicke encephalopathy Adapted from Galvinet al. [9], acquired immunodeficiency syndrome.

Clinical condition		%
Cancer		18.1
Gastrointestinal surgery		16.8
Hyperemesis gravidarum		12.2
Starvation/Fasting		10.2
Gastrointestinal tract diseases		7.7
Acquired immunodeficiency syndrome (AIDS)		5.0
Malnutrition		4.2
Dialysis and renal diseases		3.8
Parenteral nutrition		3.8
Vomiting		2.4
Psychiatric diseases		2.4
Stem cell/marrow transplantation		2.2
Other causes: infections, intoxication, thyroid disease, iatrogenic, unbalanced diet, hypoxic encephalopathy, diarrhea, unknown		0.3-3

**Figure 4:**
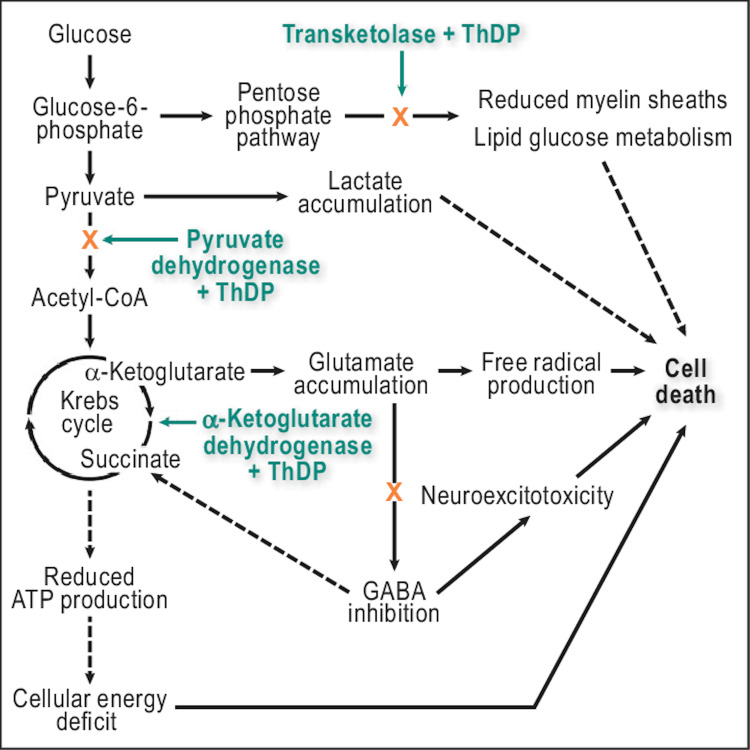
Highlighting the three thiamine-dependent enzymes and their role in cellular death as seen in thiamine deficiency. The dashed lines represent the indirect pathways. ATP: Adenosine triphosphate, CoA: Co-enzyme A, GABA: Gamma-aminobutyric acid, ThDP: Thiamine diphosphate Adapted from Jung et al. [13].

Thiamine is an important vitamin in the maintenance of cellular integrity and osmotic gradients across cellular membranes, its major storage is within bodily tissues as thiamine diphosphate (TDP). TDP is an integral cofactor for enzymes in the Krebs cycle and pentose phosphate pathway [8,10-12]. Thiamine-dependent enzymes function as a connection between glycolytic and citric acid cycles, and a deficiency in thiamine leads to an accumulation of lactate and pyruvate (Figure 4).

Thiamine deficiency also increases concentrations of alanine and glutamate as well as reducing cellular pH. This disrupts the blood-brain barrier, resulting in both cytotoxic and vasogenic edema [8,14,15]. The clinical manifestation of this is based on the fact that certain areas of the brain are vulnerable to thiamine deficiency, due to increased requirements. Affected areas include the oculomotor nerve, abducens nerve, cerebellum, hypothalamus, and diencephalon-hippocampal circuits, with the result of impairment in memory, balance, and ophthalmoplegia [5,6].

If left untreated, WE can progress to irreversible brain damage, known as Korsakoff syndrome. The hallmark of Korsakoff syndrome is confabulation, which has been attributed to anterograde and retrograde amnesia and is associated with the destruction of the bilateral mammillary bodies. When both spectrums of disease manifest simultaneously, it is known as Wernicke-Korsakoff Syndrome [6,8,16].

WE is often missed in up to 80% of cases, as it is often difficult to differentiate between alcohol intoxication or withdrawal, sepsis, hepatic encephalopathy, and head injury. WE is primarily a clinical diagnosis. In this regard, the best approach for a correct diagnosis is a high index of suspicion, good history, and physical examination skills. To aid in the diagnosis, the European Federation of Neurological Society 2010 has suggested that WE should be suspected in alcoholics with two of the following: (1) nutritional deficiency and a history of an alcohol use disorder, or any other deficiency states, (2) oculomotor abnormalities, (3) equilibrium disorders, and (4) either an altered mental state or mild memory impairment [9,17]. The presumptive diagnosis of WE can be confirmed by determining blood thiamine concentrations or by measuring red blood cell transketolase activity; however, these measurements are limited by a lack of specificity and are not readily available [11].

Imaging studies are generally not recommended as diagnostic tools for this condition but can be useful in ruling out other differential diagnoses. About the type of imaging, computed tomography (CT) can show reduced attenuation in the peri-aqueductal gray matter and the medial portion of the thalami, however, in the acute phase of WE, CT findings can be negative and thus not be used to rule out WE. If imaging is pursued, magnetic resonance imaging (MRI) is preferred because of its high specificity of 93%; however, it has a low sensitivity of 53% and thus cannot be used to exclude WE [9,17]. Usual MRI findings include a symmetrically increased T2 FLAIR signal involving the mammillary bodies, dorsomedial thalami, tectal plate, peri-aqueductal area, and/or around the third ventricle [18]. As seen in our case, there was an increased T2 signal affecting the peri-aqueductal area and bilateral mammillary bodies. 

Early treatment with thiamine is imperative as WE is reversible and timely replacement can prevent the progression to Korsakoff syndrome. There is no consensus on the optimal dosing, preparation, or duration. Pharmacokinetic studies show a blood half-life of 96 minutes, so it has been postulated that thiamine should be administered twice or thrice daily [8]. According to case report studies, WE in non-alcoholics has been cured with doses of 100mg-200 mg given intravenously; however, alcoholic patients with WE may require higher doses such as 500mg three times daily, which is recommended. In patients with WE, care must be taken not to give dextrose if patients are hypoglycemic, as this can exacerbate symptoms of WE if thiamine has not yet been repleted [8,9]. Serum magnesium should also be monitored in patients with suspected WE as it plays a role in the activity of thiamine-dependent enzymes, and, if levels are low, thiamine supplementation can be ineffective. Therefore, magnesium correction should be included as part of the treatment plan of WE [6,8,16]. 

## Conclusions

WE has many potential etiologies broadly classified as alcohol-related or non-alcohol-related. The classic triad of WE described as acute confusion, ataxia, and ophthalmoplegia is rare and is only seen in one-third of cases; our patient had all three signs of WE. This condition is grossly under-recognized and if left untreated can progress to irreversible dysfunction in Korsakoff syndrome and even death. WE is a clinical diagnosis; in this regard, high clinical suspicion, good history taking, and physical examination are required to make the diagnosis. Imaging studies are not required; however, they can help exclude other differential diagnoses. If imaging is pursued, MRI is preferred to CT due to increased specificity; however, they both lack sensitivity in the acute phase of WE. Thus, either imaging cannot be used to exclude the diagnosis. Serum thiamine levels can further confirm the diagnosis, less common is testing for red blood cell transketolase activity due to availability and reliability. The treatment of WE is with high-dose parenteral thiamine and magnesium replacement if warranted. 
